# Visual and Refractive Outcomes of Cataract Surgeries Performed in One Year in a Private Practice Setting: Review of 2714 Procedures

**DOI:** 10.1155/2020/2421816

**Published:** 2020-04-14

**Authors:** Laureano A. Rementería-Capelo, Jorge L. García-Pérez, Juan Gros-Otero, Aida Morán, José M. Sánchez-Pina, Inés Contreras

**Affiliations:** ^1^Clínica Rementería, Madrid, Spain; ^2^Hospital Universitario Ramón y Cajal, Instituto Ramón y Cajal de Investigaciones Sanitarias (IRYCIS), Madrid, Spain

## Abstract

**Methods:**

Our center's database was used to identify all isolated cataract procedures performed during 2017. The electronic records were reviewed to collect the preoperative information, presence of intra- or postsurgical complications, and visual and refractive outcomes one month after surgery.

**Results:**

In 2017, 2714 eyes of 1543 patients underwent cataract surgery in our center. Mean patient age was 70.42 years. 775 eyes (28.55%) had prior ophthalmic pathologies, and 113 eyes (4.16%) had undergone previous surgical procedures. Surgical complications developed in 35 eyes (1.29%), including 9 posterior capsule tears (0.33%) and 3 cases of dropped lens fragments (0.11%). A toric or multifocal intraocular lens was implanted in 45.6% of eyes. As regards postoperative complications, 59 eyes (2.17%) required a return to the operating theater, including 29 eyes (1.07%) requiring reinterventions due to an unexpected refractive result. There were no cases of endophthalmitis. Mean LogMAR-corrected distance visual acuity (CDVA) improved from 0.25 (SD 0.34) preoperatively to 0.04 (SD 0.17) postoperatively; 86.5% of eyes achieved a CDVA ≤0.0, with 97.5% achieving ≤0.3. In 86.4% of eyes, the difference between target and residual spherical equivalent difference was of 0.50 D or lower; 88% of eyes had a spherical equivalent ±0.50 D.

**Conclusions:**

The visual and refractive outcomes of cataract surgery in a private practice setting were excellent, well over the benchmarks set by the ESCRS. The safety profile was also within expected standards. This study provides information for ophthalmologists in private practice on expected outcomes.

## 1. Introduction

In all areas of medicine, evaluating outcomes is important for many reasons. Specifically, publication of surgical outcomes is vital for quality improvement and can help patients take decisions about their care. Studying their own outcome data can let surgeons know how they are doing compared with their peers worldwide and learn in what areas they must improve [1]. It follows that outcome data from different countries and settings should be published so that physicians do have an available standard with which they can compare themselves.

In many countries, cataract surgery has become an elective surgical procedure which patients undergo in order to become spectacle-free [2]. Advances in technical equipment, surgical procedures, and lens design have improved outcomes, but complications can still develop sometimes. Several cataract registries have been established worldwide [3]. The International Consortium for Health Outcomes Measurement (ICHOM) formed a working group to develop a global standard set of outcomes for cataract surgery, which would allow a comparison between countries [4]. Most registries include data from national health systems. The purpose of our study was to evaluate cataract surgery outcomes in a private practice setting, which may include a spectrum of patients different from those who attend a national health service.

## 2. Methods

This was a retrospective, descriptive study which aimed to report the visual and refractive outcomes, as well as the rate of complications, of isolated cataract procedures performed during one year in our center. Our clinic's database was used to identify all isolated cataract surgeries performed in the year 2017. Combined surgical procedures, such as scheduled phacovitrectomy or combined glaucoma and cataract procedures, were excluded from the study. Refractive lens exchange procedures (that is, clear lens phacoemulsification) were included, as well as those cataract surgeries that were combined with an intravitreal injection. The electronic records of all patients were reviewed in order to collect the data recommended by the ICHOM consortium. The institutional review board approval was obtained. The study followed the tenets of the declaration of Helsinki.

In our clinic, all candidates for cataract surgery undergo an extensive preoperative evaluation, including corrected distance visual acuity (CDVA), slit-lamp examination before and after pharmacological mydriasis, corneal topography, endothelial cell count, optical biometry (with ultrasonic biometry if optic signal does not have sufficient quality), fundus examination, and optical coherence tomography of the macula and optic nerve head. Candidates to toric intraocular lens implantation are also examined with the VERION™ Image Guided System (Alcon Laboratories, Fort Worth, USA). Preoperative data collected included age at the time of surgery, gender, and CDVA within one month of the surgical procedure. The presence of ocular comorbidities was recorded, including glaucoma, macular degeneration, diabetic eye disease (retinopathy and/or macular edema), amblyopia, and any other diagnosis likely to affect the visual outcome. Prior ophthalmic interventions, such as corneal refractive surgery, vitrectomy, or any other prior intervention, that might affect the outcome was also recorded, as well as whether the fellow eye had already undergone cataract surgery. Target refraction spherical equivalent was also recorded, since emmetropia was not always desired, as well as the type of intraocular lens (IOL) implanted.

Intraoperative information recorded was as follows: the surgical technique intended at the outset of the operation, technical factors such as the presence of dense, brown or white cataract, corneal opacities, pseudoexfoliation, pupillary problems (miosis, floppy iris syndrome) and any complications that occurred, including posterior capsule tears, zonular dehiscence, vitreous prolapse, dropped lens fragment into vitreous, or any other event.

All patients undergoing cataract surgery in our center are systematically followed for at least one month. Therefore, the visual outcome data provided in this study are those recorded at the one-month visit. However, preoperative and intraoperative information for all cataract surgeries performed in 2017 was included, even if for any reason the patients did not come for the one month visit. In these cases, data from the latest postoperative visit was included (first day or first week). Uncorrected and corrected distance visual acuities were recorded, as well as subjective refraction.

However, most patients keep coming to our clinic for their eye care beyond the first month, especially if there have been any complications. Therefore, any complication which developed in these patients, even beyond the one month visit, was recorded and included in the study. Any return to the operating theater caused by an intra- or postoperative complication was recorded, as well as the development of endophthalmitis, persistent corneal edema, or any other postoperative complication requiring treatment or compromising outcome.

The ICHOM recommends recording the patient reported visual function with a questionnaire; however, in 2017, our center had not included this in our practice and therefore we could not include it in our study.

Visual acuity was measured with decimal charts; it was converted to LogMAR notation prior to analysis. A visual acuity of counting fingers was assigned a LogMAR value of 2 and hand movement a value of 2.3. Statistical analysis was performed with the SPSS for Windows V.20.0 (SPSS Inc, Chicago, IL).

## 3. Results

In 2017, 2714 eyes of 1543 patients underwent isolated cataract procedures in our clinic and were included in the study. Of these, 1168 patients underwent bilateral, sequential surgery. Out of the 375 patients in whom cataract surgery was only performed in one eye in 2017, 162 (43.2%) had previously undergone surgery in the fellow eye. There were 595 men (38.6%) and 948 women (61.4%). Mean age at the time of surgery was 70.42 years (standard deviation (SD) 9.65 years), with a range from 32 to 96 years. The mean age of patients who received a multifocal IOL was lower than that of patients receiving a monofocal IOL (66.63 ± 8.97 years versus 72.19 ± 9.44 years).  Table 1 provides more details on the age of patients.

A total of 775 eyes (28.55%) had prior pathologies that might compromise visual outcomes, including 6.5% of eyes with glaucoma, 6.2% of eyes with macular degeneration, 4.1% of eyes with Fuchs corneal dystrophy, and 3.4% of eyes with amblyopia.  Table 2 records the different ocular comorbidities present prior to cataract surgery. Previous surgical procedures had been performed in 113 eyes (4.16%): 53 eyes (1.95%) had undergone laser corneal refractive surgery, 27 eyes (0.99%) vitrectomy, 12 eyes (0.44%) intravitreal injection of an antivascular endothelial growth factor (anti-VEGF) drug, 9 eyes (0.33%) pterygium excision, 6 eyes (0.22%) radial keratotomy, 5 eyes (0.18%) glaucoma surgery, and one eye strabismus surgery. As regards factors that increase surgical difficulty, 301 eyes (11.1%) had at least one risk factor, 72 eyes (2.7%) had dense or white cataracts, 28 eyes (1%) had corneal opacities, 106 eyes (3.4%) pseudoexfoliation, and 144 eyes (5.3%) pupillary problems.

The surgical procedures were performed by 14 surgeons, who had between one and eleven years of experience in cataract surgery after their residence period. Of the procedures included in this study, four surgeons performed less than 100 procedures, 4 surgeons between 100 and 200 procedures, 3 surgeons between 200 and 300 procedures, and 3 surgeons more than 300 procedures. Femtosecond laser-assisted cataract surgery (capsulotomy and lens fragmentation with the LenSx Laser, Alcon, Fort Worth, USA) was planned in 299 eyes (11.01%) but performed in only 276 cases (10.2%); the laser procedure could not be performed in 23 eyes due to insufficient pupillary dilation. Extracapsular surgery was planned and performed in only one eye, under retrobulbar anesthesia. All other procedures were planned as phacoemulsification. Surgery was performed under topical and intracameral anesthesia, through a 2.2 mm clear corneal incision. The same type of peristaltic phacomachine was used in all cases (Centurion® Vision System, Alcon laboratories, Fort Worth, USA). Each surgeon employed their preferred technique: divide and conquer, stop, and chop or prechop. At the end of the procedure, cefuroxime was injected into the anterior chamber (if patients reported known allergy to penicillin or cephalosporins, moxifloxacin was employed). As part of the prophylaxis of endophthalmitis, 5% drops povidone was applied at least one minute before starting surgery and at the end of it. Postoperative treatment was combined tobramycin and dexamethasone three times daily for one week and bromfenac twice daily for three weeks unless otherwise prescribed by the surgeon.

Surgical complications developed in 35 eyes (1.29% of procedures). In 11 of these eyes, there was at least one factor increasing the risk of complications (dense, white or polar cataract, corneal opacity, pseudoexfoliation, or pupillary problems). There were 9 posterior capsule tears (0.33%), 11 cases of zonular dehiscence (0.40%), 9 cases of vitreous prolapse (0.33%), and 3 cases of dropped lens fragments in the vitreous (0.11%). Other complications were anterior capsule tears (5 eyes, 0.18%), broken intraocular lens (IOL) or IOL haptics (4 eyes, 0.15%), misdirection syndrome (4 eyes, 0.15%), or iris bleeding (2 eyes, 0.07%). Thus, the total number of capsule complications (posterior tears, zonular dehiscence, and radial tears) was 0.92%. In one of the cases of misdirection, which occurred immediately after paracentesis, with the injection of intracameral anesthesia, surgery was delayed and performed later. Therefore, visual outcomes are provided for 2713 eyes.

The type of IOL implanted is recorded in Table 3. Almost half the eyes (45.6%) that underwent surgery in 2017 received a “premium” IOL, that is, a toric or multifocal IOL. This reflects the population that attends a private practice clinic, with a high number of patients searching for spectacle independence. The spherical power of the IOLs implanted ranged from −5.00 to +33.00 diopters (D).

As regards postoperative complications, 59 eyes (2.17%) required a return to the operating theater. Of these, 29 eyes (1.07%) required reinterventions due to an unexpected refractive result. Sixteen eyes underwent corneal refractive surgery due to residual refraction, representing 1.29% of the 1239 eyes in which a premium IOL was implanted. Nine eyes required intrasurgical rotation of a toric IOL, representing 1.4% of the 640 toric IOLs implanted. An extended depth of focus IOL was explanted due to the patient being unable to achieve neuroadaptation (0.1% of eyes of the 935 multifocal IOLs implanted). The IOL was exchanged for another in 3 eyes (0.11%) due to a refractive surprise.

Three eyes (0.11%) underwent vitrectomy to remove lens fragments from the vitreous cavity, and a further 3 eyes (0.11%) underwent delayed IOL implantation in the sulcus or anterior chamber. Five eyes (0.18%) developed a retinal detachment: 3 eyes within one month of cataract surgery and 2 eyes five and nine months after surgery, respectively; all underwent vitrectomy. Five eyes (0.11%) had cortex remains in the anterior chamber that required surgical removal and 9 eyes (0.33%) required suturing the corneal wound. Two eyes (0.07%) required surgical reposition of a subluxated IOL, one eye had to undergo surgery for the removal of posterior synechiae which developed due to an anterior segment toxic syndrome, one eye underwent vitrectomy due to malignant glaucoma, and the patient who had his surgery delayed because of early misdirection syndrome underwent uneventful phacoemulsification.

Postsurgical complications requiring some form of treatment developed in 620 eyes (22.84%), although most of them were mild and not compromising visual outcome. There were no cases of endophthalmitis and 3 cases of persistent corneal edema (0.11%), although none of them was severe enough to consider corneal transplantation. Increased intraocular pressure (IOP), defined as an IOP higher than 25 mmHg on day one after surgery, was present in 359 eyes (13.2%). Transient corneal edema developed in 48 eyes (1.8%) and a combination of increased IOP and transient edema in 27 eyes (1%). Corneal wound leaking that resolved with a contact lens and medical treatment was detected in 44 eyes (1.6%). There was a rebound anterior uveitis in 39 eyes (1.4%) after stopping topical anti-inflammatory drops, and postsurgical macular edema developed in 14 eyes (1.6%); all these responded well to topical corticosteroids and nonsteroid anti-inflammatory drugs. In 37 eyes (1.4%), posterior capsule opacification developed early after surgery, requiring YAG capsulotomy.

There were no significant differences between the overall rates of intrasurgical and postsurgical complications between femtosecond laser-assisted surgery and phacoemulsification (*P*=0.772 and *P*=0.163 respectively).

As regards visual results, only two patients did not come for the one month visit; data from the one-week visit was included for analysis. Mean LogMAR CDVA increased from 0.25 (SD 0.34) preoperatively, closest Snellen equivalent 20/40, to 0.04 (SD 0.17) postoperatively, Snellen equivalent 20/20.  Table 4 shows the visual and refractive outcomes one month after surgery. CDVA improved two or more Snellen lines in 1462 eyes (53.9%), remained stable in 1238 eyes (45.6%), and deteriorated by 2 or more lines in 13 eyes (0.5%). A detailed analysis was performed of the causes of visual loss. In 9 cases, CDVA dropped 2 lines: 4 were eyes with trifocal IOLs which developed posterior capsule opacification. After YAG capsulotomy, visual acuity improved to 20/20; however, this is not reflected in the results herein since laser was performed after the one month visit. In one case, there was a residual refractive error: visual acuity in this eye, which had also received a trifocal IOL, improved after corneal refractive surgery to 20/20. In four eyes, two implanted with a trifocal IOL and two with monofocal IOLs, postoperative CDVA reached only 20/32 for no clear reason. Of the other four cases of visual loss, two were eyes with prior dry age-related macular degeneration which developed choroidal neovascularization after surgery; CDVA dropped two and four lines, respectively. The two other cases were due to surgical complications. In a patient with myopic chorioretinopathy, there was a posterior capsular tear, with vitreous loss and sulcus IOL implantation, with postoperative hemorrhagic choroidal detachment. CDVA fell from counting fingers to hand motion. And, in an 87-year-old female, there was a posterior capsular tear, with dropped lens fragments in the vitreous. Vitrectomy with the anterior chamber iris claw IOL implantation was performed but severe anterior chamber inflammation ensued. CDVA dropped from 20/100 to hand movement, and the patient died soon after vitrectomy, due to chronic heart failure.

**Figure 1:**
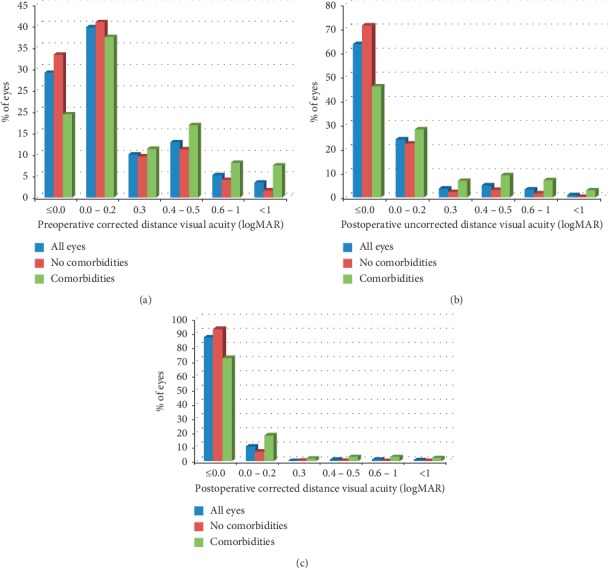
Distribution of (a) preoperative corrected distance visual acuity, (b) postoperative uncorrected distance visual acuity, and (c) postoperative corrected distance visual acuity.

Figure 1 shows CDVA distribution before and after surgery in patients with and without ocular comorbidities. Overall, 86.5% of eyes achieved a CDVA ≥20/20, with 97.5% achieving a CDVA ≥20/40. In eyes with no ocular comorbidities, the percentage of eyes achieving a CDVA ≥20/20 increased to 92.6%, with 99.7% of eyes achieving a CDVA ≥20/40.

**Figure 2:**
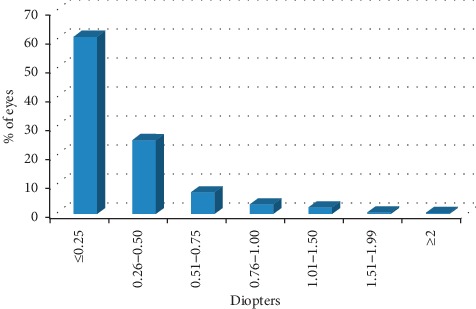
Distribution of absolute difference between target and residual spherical equivalent.

**Figure 3:**
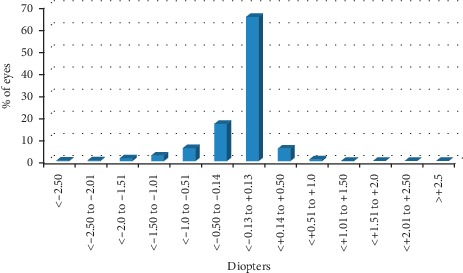
Distribution of postoperative spherical equivalent.

Refractive outcomes are recorded in Table 4.  Figure 2 shows the absolute differences between target and residual spherical equivalent for all eyes: in 86.4% of eyes, the difference was of 0.50 D or lower. There were 7 eyes in which the difference between target and residual spherical equivalent was of ≥2 D. These eyes included 3 eyes with pathologic myopia, 3 eyes with amblyopia, and 1 eye with a corneal leucoma.  Figure 3 shows postoperative residual spherical equivalent: 88% of eyes had a spherical equivalent between −0.50 and +0.50 D; 1748 eyes (64%) reached emmetropia.

A subanalysis was made of the 935 eyes which received a multifocal IOL (trifocal, bifocal and extended depth or range).  Table 5 shows visual and refractive outcomes of these eyes, and Table 6 reports on uncorrected binocular visual acuities of these patients.

## 4. Discussion

Reporting outcomes after cataract surgery is important in order to establish benchmarks for one of the most frequently performed surgical interventions worldwide [1, 5]. The advances in data collection and analyses have led to the creation of several registries, such as the European Registry of Quality Outcomes for Cataract and Refractive Surgery (EUREQUO) and the American Academy of Ophthalmology Intelligent Research in Sight (IRIS) Registry. Landmark studies have been the EUREQUO report on the results of 368 256 cataracts performed in 15 European countries [6], the EUROQUO report focused on refractive outcomes in 282 811 procedures [7], or the more recent IRIS report on the rates of endophthalmitis in 8 542 838 cataract surgeries performed in the United States [8]. However, most of the cases included in these registries do not come from private practice settings and patient characteristics, and expected outcomes may be different. The aim of this study was to report the outcomes of a private practice setting.

Patients included in our study were slightly younger, mean of 70.42 years, than those included in the 2013 (mean 73.9 years) and 2018 (mean 73.5 years) EUREQUO reports [6, 7], with a majority of women. The percentage of eyes with prior pathologies and preoperative conditions increasing the surgical complexity (28.55% and 11.1% respectively) was similar in our study to those in the previous reports [6, 9, 10].

Intrasurgical complications in our study were rare. The rate of capsular tears was 0.33%, compared with reported rates as low as 0.55% [9], to more commonly reported rates of between 1.14% and 1.78% [10–13] or occasionally as high as 3.1% [14]. Vitreous loss in our study appeared in 0.33% of cases, compared with reported rates ranging from 0.34% [9] to 2.2% [14]; the incidence of zonular dehiscence (0.40%) and dropped nucleus (0.11%) is also similar or lower compared with that of other reports [10, 14]. The low rate of intrasurgical complications in our study could be due to several factors: surgery was never performed by ophthalmologists in training and the cases included were operated recently (2017) and outcomes have been reported to improve with time [14–16].

We had no cases of postoperative endophthalmitis, taking into account the number of surgeries performed; this represents a rate lower than 0.037%. This compares favorably with the 0.05% rate reported both by the IRIS registry for 2017 [8] and by a French study for 2014 [17, 18]; a publication that analysed 21 501 eyes operated in an office setting also reported no cases of endophthalmitis [9]. This is probably due to the systematic use of topical povidone before and after surgery, together with an intracameral antibiotic at the end of the procedure. In patients with known allergy to cefuroxime, intracameral moxifloxacin was employed, which has also been shown to decrease the incidence of endophthalmitis [13].

Postoperative uveitis developed in our study in 1.4% of eyes, postsurgical macular edema in 1.6% of eyes, and retinal detachment in 0.11% of eyes. Once again, these results are similar to rates previously reported of 1.53%–2.6% for uveitis [9, 10], 0.03%–2% for macular edema [9, 10, 18], and 0.14–0.21% for retinal detachment [9, 11, 19, 20].

Preoperative CDVA was better in our study (20/40) than that in the previous reports [8]. In fact, 78.5% of eyes had a preoperative CDVA ≥20/40, with 28.9% of eyes having a preoperative CDVA ≥20/20. We have included all eyes that underwent phacoemulsification in our center in 2017, and as a private practice clinic, many patients undergo clear lens surgery as a refractive procedure. This also means that, in many cases, there was no option for visual acuity improvement in our series; thus, CDVA improved in 53.9% and remained stable in 45.6% of eyes. CDVA deteriorated in very few patients, representing 0.5% of operated eyes. This is lower than that in the previous reports; for example, the EUREQUO report found that visual acuity decreased in 1.7% of eyes [6]. A postoperative CDVA ≥20/20 was achieved in 86.5% of eyes, a much higher percentage than that previously reported (31.31%–61.2% [6, 8]), with only 2.6% not reaching 20/40 (previous reports 5.8% [6]). Mean CDVA after surgery in our study (0.04 ± 0.17) is much more similar to Ianchulev et al.'s office-based report, with a CDVA of 0.14 ± 0.26 [9]. Lundström et al. showed decreasing visual thresholds for surgery, decreasing surgical complication rates, and increasing visual outcomes regardless of the initial preoperative visual level [16].

We believe there may be several reasons explaining our better results: first of all, the procedures reported herein were performed in 2017, and as already mentioned, it has been shown that surgical outcomes improve with time. We have no residents in our center, and therefore the incidence of surgical complications was lower than in a teaching hospital. Before surgery, a thorough ophthalmological examination is always performed, in which any factors which may complicate surgery are identified. And finally, refraction and visual acuity measurements after surgery were always performed by experimented optometrists.

As regards refractive outcomes, our results also compare favorably with the recent reports. Mean absolute error between target and postoperative spherical equivalent was 0.28 D (SD 0.31 D), with 86.4% of eyes within 0.50 D of target. A study on 8943 cataract procedures performed in the United Kingdom between November 2006 and December 2016 found a mean absolute error of 0.50 D (SD 0.46), with 62.36% within 0.5 D of target [21], and the EUROQUO report on refractive outcomes including surgeries performed in 2014 and 2015 found a mean absolute biometry prediction error of 0.42 D (SD 0.52) and 72.7% of eyes within 0.5 D of target [7]. Patients implanted with multifocal IOLs expect to be spectacle independent, and in order to achieve this and to optimize IOL performance, postoperative refraction should be close to emmetropia. We therefore did a subanalysis of eyes that had received a multifocal IOL. A recent big-data study analysed the outcomes of 10 084 trifocal IOLs in 5048 patients (5 802 FineVision IOLs and 4 282 AT Lisa tri IOLs) 3months after surgery in our country [22]. Although in our study, visual acuity and refraction were measured at 1month, and to establish comparisons is difficult, the postoperative uncorrected distance visual acuity of the eyes in our study, 0.11 (SD 0.23) is only slightly lower than that reported for the AT Lisa Tri (0.04 (SD 0.08)) and the Finevision (0.06 (SD 0.08)) and CDVA in our study 0.04 (SD 0.17), very similar: 0.02 (SD 0.06) for the AT Lisa Tri and 0.03 (SD 0.06) for the Finevision. Mean residual spherical equivalent was −0.16 (SD −0.48) in our study, compared with 0.26 (SD 0.47) for the AT Lisa Tri and 0.34 (SD 0.50) for the Finevision. These excellent refractive outcomes may be due to several factors. Corneal topography is performed systematically, and patients with corneal astigmatism receiving toric IOLs are also examined with the VERION™ Image Guided System, which is also used to guide IOL implantation. The IOL is chosen together by the surgeon and a team of experimented optometrists.

Limitations of this study are its retrospective nature and the fact that no patient-reported outcomes were collected. For a surgical procedure like cataract surgery, which nowadays has such a low rate of complications, it is important to meet patient expectations and therefore it is vital to record and evaluate patient perception. The problem is that patients are followed only for one month or less in routine practice and the full import of visual improvement, need for spectacle correction, and adaptation to dysphotopsias are only correctly perceived several months after surgery. Maybe a further follow-up visit should be always scheduled between three and six months after cataract surgery in order to evaluate the patient perception.

In summary, we have found excellent safety results for cataract surgery in a private practice setting, with outstanding visual and refractive outcomes. This study supports the notion that the safety and efficacy of this procedure are constantly improving and that it may be adequate to review the benchmarks that have been accepted up to date.

## 5. Conclusions

Evaluating outcomes after cataract surgery is important in order to compare each practice's results with established benchmarks. Cataract surgery in our private practice setting has excellent visual and refractive outcomes, well over the benchmarks provided by the European registry. CDVA deteriorated by 0.2 or more in 13 eyes (0.5%) at the one month visit, but in only two cases was this due directly to surgical complications. Premium intraocular lens were implanted in almost half the patients who underwent surgery in 2017, reflecting the increasing desire for spectacle independence.

## Figures and Tables

**Table 1:** Ages of the patients included in the study.

Years	Number of patients (percentage)
≤40	4 (0.3%)
41 to 50	44 (2.9%)
51 to 60	196 (12.7%)
61 to 70	474 (30.7%)
71 to 80	609 (39.5%)
81 to 90	201 (13%)
>90	15 (1%)

**Table 2:** Ocular comorbidities prior to cataract surgery.

	Percentage (number of eyes)
Glaucoma	6.5% (177)
Macular degeneration	6.2% (169)
Fuchs corneal dystrophy	4.1% (112)
Amblyopia	3.4% (93)
RPE changes and drusen^*∗*^	3.2% (86)
Epiretinal membrane, lamellar macular hole, or vitreomacular traction	2.5% (64)
Myopic chorioretinopathy	2.5% (68)
Corneal leucomas	1% (28)
Previous retinal detachment surgery	0.6% (15)
Neuro-ophthalmologic pathology	0.6% (16)
Diabetic eye disease	0.4% (12)
Macular scar	0.3% (7)
Previous macular hole or epiretinal membrane surgery	0.3% (9)
Others: stargardt disease, pigmentary retinosis, retinal vein occlusion, queratoconus, macular hole, iris and retinal coloboma, uveitis, myopic choroidal neovascularization	0.8% (22)

^*∗*^Retinal pigmentary epithelium changes and drusen that were not classified as macular degeneration by the attending physician but that were described in the preoperative assessment.

**Table 3:** Type of intraocular lens implanted.

	Percentage (number of eyes)	Models used (number of eyes)
Monofocal, spheric	54.2% (1472)	Alcon MN60AC (7), Alcon MN60MA (43), Alcon SA60AT (25), Alcon SN 60WF (1357), Bausch & Lomb Envista (40)
Monofocal, toric	11.1% (302)	Alcon SN6ATX (228), Bausch & Lomb Envista Toric (22), Ophtec Precizon Toric (52)
Trifocal	19.6% (533)	Alcon Panoptix TFNT00 (475), Physiol Finevision Micro F (16), Physiol Finevision PodF (42)
Trifocal, toric	11.5% (313)	Alcon Panoptix TFNTXX (244), Physiol Finevision Pod FT (67), Carl Zeiss AT Lisa Tri 839 MP (2)
Extended depth of focus, spheric	1.4% (39)	Johnson & Johnson Tecnis Symfony ZXR00 (39)
Extended depth of focus, toric	1.3% (35)	Johnson & Johnson Tecnis Symfony ZXTXX (35)
Bifocal, spheric	0.6% (15)	Carl Zeiss AT Lisa 809M (8), Johnson & Johnson Tecnis Symfony ZLB00 (6), Topcon Lentis LS-313MF (1)
Anterior chamber, spheric	0.2% (4)	Ophtec Artisna Aphakia (3) Alcon MTA4U0 (1)

**Table 4:** Visual and refractive outcomes one month after cataract surgery for all eyes.

Visual acuity	
Preoperative corrected distance visual acuity	0.25 (SD 0.35)
	Range 2.30 to −0.10
Postoperative uncorrected distance visual acuity	0.11 (SD 0.23)
	Range 2.30 to −0.20
Postoperative corrected distance visual acuity	0.04 (SD 0.17)
	Range 2.30 to −0.20
Change in corrected distance visual acuity	−0.22 (SD 0.03)
	Range 1.60 to −2.30

*Refraction*	
Target spherical equivalent (D)	−0.27 (SD 0.43)
	Range from −3.82 to 1.84
Residual spherical equivalent (D)	−0.16 (SD −0.48)
	Range from −3.50 to 4.25
Difference between target and residual spherical equivalent (D)	0.11 (SD 0.41)
	Range from −3.27 to 4.95
Absolute vale of difference between target and residual spherical equivalent (D)	0.28 (SD 0.31)
	Range from 0 to 4.95

D: diopters. Visual acuity is reported as LogMAR value

**Table 5:** Visual and refractive outcomes one month after cataract surgery of eyes receiving a multifocal intraocular lens (*n* = 935).

Visual acuity	
Preoperative corrected distance visual acuity	0.16 (SD 0.27)
	Range 2.0 to −0.10
Postoperative uncorrected distance visual acuity	0.04 (SD 0.09)
	Range 0.50 to −0.20
Postoperative corrected distance visual acuity	0.01 (SD 0.05)
	Range 0.30 to −0.20
Change in corrected distance visual acuity	−0.15 (SD 0.27)
	Range 0.2 to −2.0

*Refraction*	
Target spherical equivalent (D)	−0.27 (SD 0.43)
	Range from −3.82 to 1.84
Residual spherical equivalent (D)	−0.04 (SD −0.22)
	Range from −1.88 to 0.75
Difference between target and residual spherical equivalent (D)	0.08 (SD 0.27)
	Range from −1.85 to 1.33
Absolute vale of difference between target and residual spherical equivalent (D)	0.21 (SD 0.19)
	Range from 0 to 1.85

D: diopters. Visual acuity is reported as LogMAR value.

**Table 6:** Binocular uncorrected visual acuities of patients implanted with multifocal intraocular lenses.

Binocular uncorrected visual acuity	Trifocal/bifocal (451 subjects)	Extended depth of focus (38 subjects)
Distance	0.00 (0.05)	0.01 (0.06)
	Range 0.40 to −0.10	Range 0.30 to −0.10
Intermediate	0.12 (0.10)	0.13 (0.11)
	Range 0.50 to 0.00	Range 0.40 to 0.00
Near	0.01 (0.05)	0.10 (0.16)
	Range 0.40 to 0.00	Range 0.70 to 0.00

Intermediate visual acuity was measured at 66 cm and near visual acuity at 40 cm.

## Data Availability

The data analysed during the current study are available from the corresponding author on reasonable request.
